# Lupus pathobiology based on genomics

**DOI:** 10.1007/s00251-016-0961-7

**Published:** 2016-12-08

**Authors:** Mohammad Saeed

**Affiliations:** Arkana Laboratories, 10810 Executive Center Drive, Suite 100, Little Rock, AR 72211 USA

**Keywords:** Lupus, Genomics, Therapeutics, Immunology

## Abstract

Systemic lupus erythematosus (SLE) is an autoimmune disorder with complex genetic underpinnings. This review attempts to assemble the myriad of genomic findings to build a clearer picture of the pathobiology of SLE to serve as a guide for therapeutics. Over 100 genes are now known for SLE, and several more penetrant ones have led to the emergence of more defined lupus phenotypes. Also discussed here are the targeted therapies that have come up on the horizon and the specific biologic mechanisms of more traditional therapies which have only recently been explored. The diagnostic toolbox has been enhanced by the addition of new antibodies, gene expression signatures, and mutation panels. This provides an opportunity to piece together the lupus puzzle and even revisit the clinical classification of SLE.

## Introduction

Lupus is a syndrome rather than a single disease. Based on the ACR criteria 1997, there are 330 types of lupus possible [11!/(4! × 7!)]. This phenotypic variability makes every lupus patient unique and uniform clinical classification difficult. Consequently, molecular pathobiology of lupus, which has the potential for improved clinical disease definition and providing targeted therapies, becomes challenging. Lupus definition has evolved over time from exclusively dermatologic to multiorgan systemic disease (Scofield and Oates 2009). The discovery of the LE cell and antibodies to DNA in the mid-twentieth century further transformed lupus diagnosis and significantly impacted its clinical management (Hargraves et al. 1948; Deicher et al. 1959). Discovery of immunological features such as immune complex deposition in lupus nephritis (LN), low serum levels of complement components, and a variety of autoantibodies made lupus a more defined entity by early 1980s (Koffler et al. 1974; Jennette and Hipp 1985; Kunkel 1983). Since then, significant advances in the genetics of systemic lupus erythematosus (SLE) have taken place which are helping unravel the lupus enigma and will in the near future even define therapy. Here, we review the pathobiology of lupus based on genomic findings to enhance clinical diagnosis and therapeutics.

## Monogenic lupus

Rare genetic diseases provide valuable insights into more common disorders. Focused analyses in families with multiple lupus-like clinical phenotypes have led to the identification of several monogenic causes of lupus that were subsequently verified by association studies. One of the prototype monogenic pediatric lupus syndromes is Aicardi-Goutieres syndrome (AGS) characterized by encephalopathy, cerebral atrophy, basal ganglia calcifications, seizures, thrombocytopenia, hypocomplementemia, and presence of multiple autoantibodies. Due to the clinical similarity, several AGS patients have been formally diagnosed with SLE. AGS involves recessive and dominant mutations in RNAses (*RNASEH2A*, *RNASEH2B*, *RNASEH2C*), a DNAse (*TREX1*), double-stranded RNA (dsRNA) editing (*ADAR*), dsRNA recognition and binding (*IFIH1*), and activation of the innate immune system (*SAMHD1*, *IFIH1*) (Crow et al. 2015).

*TREX1* and *IFIH1* have been shown to be associated with SLE as well (Table 1). *TREX1* is a major intracellular DNAse with 3′ exonuclease activity leading to single-stranded DNA degradation during caspase-independent apoptosis, minimizing autoimmune reactivity to self-DNA. In AGS, homozygous mutations in *TREX1* associated with loss of protein activity are frequently found. In early-onset cerebral SLE, exome sequencing identified a pathogenic variant in *TREX1* (Ellyard et al. 2014) and a heterozygous mutation was shown to cause familial chilblain lupus (Günther et al. 2009). A sequencing-based association study of *TREX1* exons in four European SLE cohorts identified 12 heterozygous missense and frameshift changes in over 400 SLE patients (*P* = 1.7 × 10^−7^) (Lee-Kirsch et al. 2007). In essence, these studies have not only identified *TREX1* as a major SLE gene but also unraveled part of the lupus pathobiology where autoimmunity results from aberrant processing of DNA during apoptosis.

**Table 1:**
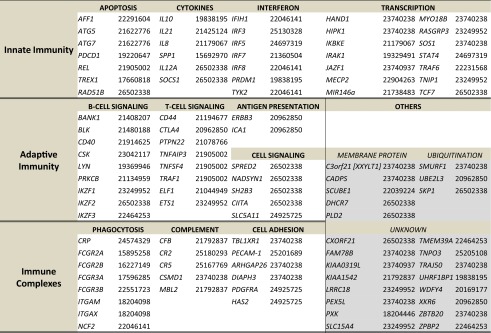
Published SLE-associated genes

Summary of genes in which variants are associated with SLE in GWAS and candidate association studies. The genes are grouped according to their pathways. Numbers in parentheses indicate study PubMed IDs for easy reference

Similarly, other DNAses have also been shown to be involved in lupus viz. *DNAse1L3*, *DNASE1*, and *POLB*. *DNAse1L3* was identified to cause familial SLE in Middle Eastern families with multiple affected children (Al-Mayouf et al. 2011). These patients had positive ANA, dsDNA, and ANCA antibodies; low C3 and C4; and high frequency of nephritis. Linkage analysis located the locus to 3p14.3, where an earlier genome-wide association study (GWAS) peak was also noted (though attributed to a nearby gene, PXK, 140 kb from *DNAse1L3*; Table 1). *DNAse1L3* sequencing found mutations which decreased protein activity or eliminated gene expression. Interestingly, it had been previously shown that *DNAse1L3* deficiency increases the susceptibility of mice to polygenic SLE (Wilber et al. 2003). Moreover, hypocomplementemic urticarial vasculitis syndrome (HUVS) which shares similarity with SLE was found to result from *DNAse1L3* mutations in two Turkish families (Ozçakar et al. 2013). Majority of these patients had positive ANA and dsDNA antibodies; low C3 and C4, class II, III, and glomerulonephritis (GN); and recurrent urticarial rash with leukocytoclastic vasculitis, fever, anemia, lymphadenopathy, and arthritis. ANCA was positive in two of five patients. Due to clinical and immunologic similarity to SLE, HUVS is considered by some as an SLE-associated syndrome (Aydogan et al. 2006). Additionally, HUVS is present in ∼8% of lupus patients, and SLE is observed in >50% of HUVS patients during follow-up (Aydogan et al. 2006).

A small sequencing study found in 2 of 20 SLE patients, a heterozygous non-sense mutation (A → G transversion at position 172 in exon 2) decreased the activity of *DNASE1*, the most abundant extracellular DNAse (Yasutomo et al. 2001). Both these patients had positive ANA, dsDNA, SSA, and RNP antibodies; fever; and generalized erythematous rash and nephritis. Finemapping of a SLE GWAS signal on 8p11.21 identified *POLB* encoding DNA polymerase beta (Pol β), which repairs single-strand DNA breaks (Sheng et al 2011). It was shown that mutant *POLB* (Y265C) repairs DNA significantly more slowly than do wild-type (WT) Pol β, and mutant *POLB* mice develop dermatitis, GN, cervical lymphadenopathy, and high titers of ANA, i.e., pathology resembling SLE (Senejani et al. 2014).

All *IFIH1* mutations in AGS are dominant gain of function, though majority of AGS is autosomal recessive. *IFIH1* codes for melanoma differentiation-associated gene 5 (MDA5), a cytoplasmic receptor that binds to viral long dsRNA structures and induces IFN-β production (Hall and Rosen 2010). *IFIH1* was shown to be strongly associated with SLE in large association studies of European patients (Cunninghame Graham et al. 2011; Gateva et al. 2009). Interestingly, MDA5 (encoded by *IFIH1*) is associated with a dermatomyositis syndrome that carries its name. MDA5 syndrome has been classified as an amyopathic (absence of muscle involvement) dermatomyositis characterized by a specific 140-kD antibody in serum (Betteridge et al. 2011), palmar papules, periungual erosions, alopecia, arthritis, and severe interstitial lung disease (ILD) with extremely poor prognosis (Fiorentino et al. 2011). About 50% of MDA5 patients die of ILD within 6 months of diagnosis (Nakashima et al. 2010). Though clinically distinct phenotypes are known to be encoded by different mutations in a single gene (Deng et al. 2010; Fecto et al. 2011), it is also possible that MDA5 is a monogenic lupus syndrome with a specific antibody rather than dermatomyositis since being clinically amyopathic is an essential feature of MDA5 or perhaps the MDA5 antibody is also present in SLE cohorts given the strong *IFIH1* association. In mice with MDA5 overexpression, there was increased interferon gene signature (IGS), resistance to lethal viral infection and when combined with a lupus susceptible background, production of autoantibodies and GN was accelerated (Crampton et al. 2012). However, in mice with a gain-of-function mutation in MDA5, a lupus syndrome developed spontaneously and type I interferon signaling was found to be crucial (Funabiki et al. 2014).

Another characteristic of AGS is elevated interferon-α (IFN-α) levels in serum and CSF as well as IGS upregulation. This is true for all AGS mutant genes including *IFIH1*. Recently, the gene *ACP5* encoding tartrate-resistant acid phosphatase (TRAP) was discovered for Spondyloenchondrodysplasia (SPENCD; MIM271550), a skeletal dysplasia syndrome with intracranial calcifications, spasticity, and immunologic abnormalities (Briggs et al. 2011). Of the ten study patients with SPENCD, four also met the ACR criteria for SLE, presenting with arthralgia/arthritis, seizures, rash, cytopenias, LN (class IV and V), hypocomplementemia, and positive ANA and dsDNA antibodies (Briggs et al. 2011). In SPENCD patients, serum IFN-α and IGS in macrophages and dendritic cells were elevated, due to loss of function mutations in *ACP5*.

We recently reported the association of apolipoprotein L1 (*APOL1*) risk alleles with SLE collapsing glomerulopathy (CG) in the largest series to date of renal biopsies from African-American patients with LN (Larsen et al. 2013). CG has uniformly poor prognosis with rapid progression to renal failure and no response to immunosuppression. We also showed that *APOL1* variants are associated with specific pathologic features such as microcystic tubular dilatation (Larsen et al. 2015). *APOL1* risk alleles were previously shown to be associated with HIV nephropathy (HIVAN). Interestingly, HIV and SLE, two pathogenically divergent processes, result in very similar glomerular pathology, i.e., CG and even proliferative GN (D’Agati and Appel 1997). Moreover tubuloreticular inclusions on electron microscopy in the glomerular endothelial cytoplasm are unique to HIV and SLE (D’Agati and Appel 1997). Normal cells develop these inclusions after exposure to elevated levels of IFN-α (Rich 1995), and CG development has been reported with pharmacologic interferon treatment (Rich 1995). This morphologic overlap is likely a clue to the pathogenesis of CG and the type of inflammatory milieu that incites disease, specifically elevated IFN levels. It also strengthens the relationship of type I interferon signaling with SLE.

Protein kinase C δ (PKCδ) encoded by the gene *PRKCD*, a serine/threonine kinase implicated in apoptosis (Mecklenbräuker et al. 2002), was shown to be linked to SLE in a family of three affected siblings (Belot et al. 2013). These patients had positive ANA and dsDNA antibodies, hypocomplementemia, rash, LN, CNS vasculitis, lymphadenopathy, and hepatosplenomegaly. The mutant PKCδ decreased apoptosis in transitional and primary B-cells (Belot et al. 2013). Similarly, mice deficient in PKCδ demonstrate a defect in the negative selection of self-reactive B-cells, an expansion of peripheral B-cells and develop features consistent with SLE (Mecklenbräuker et al. 2002). Correspondingly, an intronic variant (rs16972959) in *PRKCB*, a member of the PKC-gene family and encoding PKCβ, was found to be associated with SLE in a Han Chinese population (Sheng et al. 2011). In lupus-susceptible mice, PKCβ deficiency abolished nephritis and autoantibody formation by inducing an anergic B-cell phenotype and treatment with the PKCβ-specific inhibitor enzastaurin prevented the development of lupus (Oleksyn et al. 2013).

Hypocomplementemia is another theme that runs through lupus. Detailed discussion on complement system involvement in lupus pathogenesis has been presented elsewhere (Macedo and Isaac 2016); however, briefly early complement component deficiency leads to autoimmunity. SLE develops in over 90% of C1q-deficient individuals though complete deficiency of C1q is rare (Botto et al. 1998). Similarly, SLE development is strongly associated with C4 deficiency (75%) and to a lesser degree with homozygous C2 deficiency (10–30%) though C2 deficiency is the most common in European populations (0.5–1/10^4^) (Botto et al. 2009). Complement deficiencies were the first monogenic causes of lupus discovered (Nishino et al. 1981; Hannema et al. 1984) and also became one of the first diagnostic (Koffler et al. 1974; Schroeter et al. 1976) and subsequently predictive markers for SLE activity (Swaak et al. 1986). Presence of multiple autoantibodies, rash, arthritis, and LN are common features of SLE associated with complement deficiencies. The significance of the complement system is in clearance of apoptotic debris which is hampered by its deficiencies.

## Polygenic lupus

**Fig. 1:**
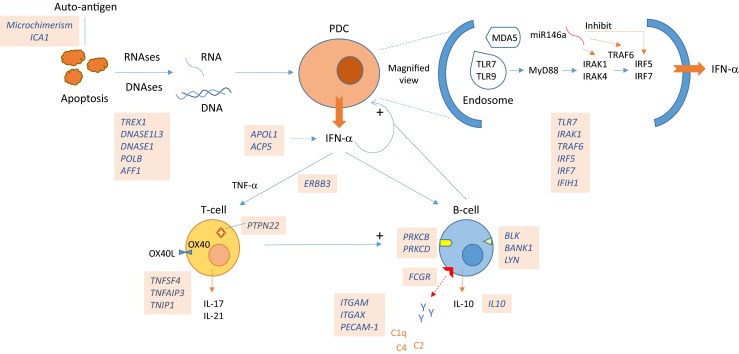
SLE pathobiology based on genomic findings. SLE initiation: aberrant apoptosis triggered by dysfunctional DNAses/RNAses activate the endosomal TLR, MDA5, and related pathways in pDCs. Inherent B-cell dysfunction and HLA-associated thymic T-cell negative selection may result in autoantibody formation. SLE potentiation: increased IFN-α, dysfunctional antigen presentation by pDCs, and inherent malfunctioning of T- and B-cells leading to a proinflammatory milieu causes persistent tissue inflammation. Consequent organ damage is exacerbated by defective IC clearance

The perpetual cycle of immunologic abnormalities that defines lupus was elucidated in 1963 and is based on aberrant apoptosis followed by immune complex formation and deposition (Sbarra et al. 1963). The recent addition of the IGS to this cycle may provide another marker of SLE activity and pathogenically be indicative of the central role of plasmacytoid dendritic cells (pDCs) which are the major producers of IFN-α. The intracellular signaling of apoptotic material in pDCs is mediated by specialized receptor systems such as MDA5 and Toll-like receptors (TLRs; Fig. 1). There are ten TLR-members in humans; e.g., TLR3 is a receptor for dsRNA; lipopolysaccharide (LPS) of gram-negative bacteria is recognized by TLR4; and TLR7 is activated by ssRNAs and TLR9 by unmethylated ssDNA (Akira et al. 2001; Panter et al. 2009). TLR signaling pathways are highly complex and mediate a variety of essential injury-and-repair processes including phosphorylation; ubiquitination; and induction of inflammatory cytokines, apoptosis, and autophagy (Allam and Anders 2008). Gene expression of TLR 2, 7, and 9 in peripheral blood mononuclear cells from SLE patients has been found to be higher than healthy controls (Komatsuda et al. 2008). Aberrant signaling of TLRs is known to trigger autoimmunity (Allam and Anders 2008) and the nucleic acid binding TLRs in particular have been implicated in SLE (Savarese et al. 2008).

The BXSB male mice develop an accelerated, lupus-like phenotype with severe nephritis (Blossom et al. 1997) due to an X-to-Y chromosomal translocation of a gene cluster known as Y autoimmune accelerator (Yaa) (Subramanian et al. 2006). Subsequently, *TLR7* was identified as the causative gene in the Yaa cluster (Deane et al. 2007) and a candidate-gene association (CGA) study reported that a functional polymorphism in 3′ UTR of *TLR7* was associated with SLE in Asian populations, with a stronger effect in male than female subjects (Shen et al. 2010).

Genes associated with SLE located on the X-chromosome may in part explain the extreme female preponderance of the disease. This concept of the X-chromosome gene dose effect in SLE is supported by the heightened prevalence of SLE in Klinefelter’s syndrome (47,XXY), shown to be 2.2%, i.e., 13-fold higher than in normal men and similar to that of normal women (Scofield et al. 2008; Dillon et al. 2012). SLE-associated genes located on the X-chromosome include *CXorf21*, *MECP2*, and *IRAK1* as well as *TLR7* and *TLR8* (Table 1). Variants in *MECP2* may disinhibit its target genes such as *IRAK1* and lead to heightened IFN signaling. *MECP2* is also involved in methylation with consequent aberrations in gene expression in SLE. Though the function of *CXorf21* is unknown, it escapes X-inactivation and the large GWAS that recently identified it also had a significantly higher prevalence of Klinefelter’s in its male cases with SLE (Bentham et al. 2015). Skewed X-inactivation in certain tissues may predispose to SLE development if the preferred parental X-chromosome harbors these or other SLE-associated genes (Weckerle and Niewold 2011).

TLR signaling leads to NFκB activation and IFN production which in turn stimulates the JAK/STAT pathway leading to release of proinflammatory cytokines. There are several SLE-associated genes involved in these pathways including *IRAK1*, *TRAF6*, *IRF5*, *IRF7*, *STAT4*, *TNFAIP3*, and *TYK2* (Table 1 and Fig. 1). MicroRNAs (miRNAs) regulate TLR and IFN signaling (Jacob et al. 2009a). They are non-coding RNAs (19–25 nucleotides long), ubiquitously expressed across evolutionary strata and regulate gene expression by inhibiting mRNA translation and targeting specific mRNAs for degradation. It has been shown that miR-146a inhibits *IRAK1*, *TRAF6*, and *IRF5 (*Jacob et al. 2009a *)*. It is upregulated by TLR ligands and IFN, thus functioning as an inducible repressor of the innate immune system (Wang et al. 2008a). Variants in miR-146a are associated with SLE and prevent the normal downstream binding of the transcription factor *ETS1*, which is also associated with SLE, leading to disinhibition of the IFN pathway (Fig. 1) (Jacob et al. 2009a; Wang et al. 2008a).

The potential triggering of SLE through TLRs, MDA5, JAK/STAT, and NFkB pathways in pDCs by viruses (e.g., RNA viruses such as picornaviruses), bacteria (LPS), and products of aberrant apoptosis (dsDNA, ssDNA, and dsRNA) has been discussed thus far. During pregnancy, fetal cell transfer across placental interface into maternal circulation, called microchimerism, could also act as a triggering antigen for SLE flares (Fig. 1) (Arck and Hecher 2013). Y-chromosome DNA can be detected by PCR in maternal blood and tissues and is indicative of microchimerism. Chimeric male cells in postmortem female organs were identified at significantly higher frequency in SLE patients than in controls (Kremer Hovinga et al. 2007). Similarly, a vaccine component may serve as an autoantigen as well. This rare phenomenon is called autoimmune/inflammatory syndrome induced by adjuvants (ASIA). Changes in ANA titers and subsets have been reported in a small number (∼5%) of SLE patients after vaccination, and there have been a few cases of SLE onset or exacerbation following vaccination (Perdan-Pirkmajer et al. 2012; Soldevilla et al. 2012). Rarity of this phenomenon points to the robustness of the immune system at dealing with foreign antigens and their low likelihood of being causative for SLE in general.

Islet cell autoantigen 1 (*ICA1*) which is associated with SLE (Table 1) was previously known to function as an autoantigen in type I diabetes mellitus (Pietropaolo et al. 1993). Recently, it was shown that polymorphisms in *ICA1* promoter may alter binding of the transcription factor *AIRE*, resulting in downregulation of *ICA1* expression in medullary thymic epithelial cells leading to loss of immunologic tolerance to this self-antigen and triggering autoimmunity (Bonner et al. 2012). Interestingly, mutations in *AIRE* lead to autoimmune polyendocrinopathy candidiasis ectodermal dystrophy (APECED) in which multiple autoantibodies form against several endocrine organs as well as against IFN, IL17, and IL22 (Kluger et al. 2012). APECED does not clinically resemble SLE, pointing to perhaps the significance of IFNs to SLE pathogenesis. Anti-IL17 response is the likely cause of candidiasis seen in APECED.

Antigen presentation by pDCs is determined by HLA-coded proteins (i.e., MHC) and subsequently leads to the generation of autoantibodies and immune complexes amplifying both innate and adaptive immune responses. The HLA region is subdivided into class I and class II regions, which contain genes encoding glycoproteins that process and present peptides for T-cell recognition and the class III region harboring complement components (*C2*, *C4A*, *C4B*, and *CFB*), *TNF*, and other immune genes. GWAS in both European and Asian populations have shown that the strongest contribution to risk for SLE resides in the HLA region and consists of multiple genetic effects (Graham et al. 2009). However, due to the long-range linkage disequilibrium (LD) of ∼1 Mb within the HLA region, the exact susceptibility gene is hard to identify. HLA DRB1*1501 (DR2) and DR3 B1*0301 are class II alleles consistently shown to be associated with SLE (International MHC and Autoimmunity Genetics Network et al. 2009). More recently, a large GWAS found that the best model for association was a combination of HLA alleles including B*08:01 and B*18:01 in class I, DQB1*02:01, DRB3*02:00, and DQA*01:02 in class II and a class III SNP (rs74290525) located in *SLC44A4* (Bentham et al. 2015). Previously, the association signal in class III had been fine mapped to the sixth intron of the *SKIVL2* gene as well as to a SNP located between *TNXB* and *CREBL1* and the HLA DRB1*0301 (International MHC and Autoimmunity Genetics Network et al. 2009).

TNF-ligand superfamily member 4 (*TNFSF4*) is expressed on pDCs and interacts with its receptor, OX40L, on activated T-cells. OX40L-mediated signaling inhibits the generation and function of IL-10-producing CD4+ T-regulatory-cells (Ito et al. 2006) but induces B-cell activation and differentiation, as well as IL-17 production (Fig. 1) (Stüber et al. 1995). Simultaneously, IFN-α is known to induce a proinflammatory gain of function of IL-10, an important pleiotropic cytokine involved in B-cell regulation (Sharif et al. 2004). Binding of miR-21 to the 3′ UTR of *RASGRP1* (Table 1) activates the Erk–MAP kinase cascade leading to the inhibition of downstream target gene *DNMT1*, a methyltransferase that is a major epigenetic regulator. Upregulation of miR-21 leads to DNA hypomethylation of CD4+ T-cells in SLE (Jacob et al. 2009a). *PTPN22* encodes a tyrosine-phosphatase that inhibits T-cell activation. The non-synonymous SNP rs2476601 associated with SLE is a gain-of-function variant that reduces the threshold for T-cell-receptor signaling and promotes autoimmunity (Kyogoku et al. 2004). Blocking T-cell activation by CTLA4-Ig prevented LN and delayed mortality in NZB/W F1 mice (Finck et al. 1994). Kidneys of nephritic NZM2328 mice show increased expression of Th1 cytokines (IFN-γ, IL-12) (Jacob et al. 2009b) whereas in MRL/lpr mice, laser microdissection of renal infiltrates showed increased Th1 and IL-17 expression (Wang et al. 2008b). Th17 expression profile resulted in TNF-receptor-deficient NZM2328 mice in an accelerated onset of LN and higher mortality (Jacob et al. 2009b). IL-17 was also found to be increased in ∼25% of SLE patients (Garrett-Sinha et al. 2008). Thus, Th1 and Th17 T-cells play a significant role in SLE and interact with pDCs and B-cells to coordinate the lupus immunologic cascade.

Several B-cell-related genes are associated with SLE (Table 1). *BLK* encodes tyrosine-protein kinase Blk, a member of the src kinases, which mediates the proliferation, differentiation, and tolerance of B-cells. B-cell scaffold protein with ankyrin repeats (*BANK1*) regulates direct coupling between the src-tyrosine kinases and the calcium channel IP(3)R facilitating intracellular calcium release and altering B-cell activation threshold (Yokoyama et al. 2002). It has been shown in mouse models that BANK1 regulates TLR7 signaling in B-cells and that B-cell innate immune response via TLR7 is critical for the development of autoimmunity (Wu et al. 2016; Walsh et al. 2012). Tyrosine-kinase lyn (*LYN*) mediates B-cell activation by phosphorylating the activation motif of the B-cell-receptor-associated Ig α/β signaling molecules and mediates B-cell inhibition by phosphorylating inhibitory receptors such as CD22 (Hibbs et al. 2002). Lyn-overexpressing mice developed autoantibodies and lethal LN (Hibbs et al. 2002). A transgenic mouse (MRL/lpr mice) expressing a unique B-cell receptor leading to lack of circulating Ig has been shown to develop LN (Chan et al. 1999). Therefore, aberrant B-cell functioning plays a much more significant role in SLE than perhaps antibody formation.

B-cell low-affinity IgG Fc-receptors (FcGRs) encoded by *FCGR2A*, *FCGR2B*, *FCGR3A*, and *FCGR3B* are involved in immune complex (IC) processing and clearance by antibody-dependent responses (Table 1). Other SLE susceptibility genes with important roles in IC processing are *ITGAM*, *ITGAX*, and complement genes. *ITGAM* codes for an integrin, important in the adherence of neutrophils and monocytes to stimulated endothelium, and also in the phagocytosis of complement-coated particles. A large GWAS comparing LN with SLE patients in an attempt to discover LN susceptibility genes found *PDGFRA* (involved in matrix synthesis, chemotaxis, and cytokine production) and Hyaluronan synthetase 2 (HAS2; also involved in extracellular matrix production) to be strongly associated (Chung et al. 2014).

There is substantial evidence that disease pathways interact to create the lupus milieu. On a large SLE dataset of European patients, gene interactions were detected between the HLA region and *CTLA4*, *IRF5*, and *ITGAM* and between *PDCD1* and *IL-21* (Hughes et al. 2012 *)*. Another study showed epistasis of *BLK*, *TNFSF4*, *TRAF1*, *TNFAIP3*, and *REL* genes in SLE (Zhou et al. 2012). Moreover, coimmunoprecipitation and colocalization experiments have shown physical interaction between *BLK* and *BANK1* (Castillejo-López et al. 2012). Similarly, epistasis has also been demonstrated in mouse models of lupus (Morel et al. 2000).

It was previously thought that specific pathogenic autoantibodies lead to target organ damage in SLE; however, passive transfer of anti-dsDNA antibodies did not lead to LN (Vlahakos et al. 1992) and immunoglobulins (Ig) eluted from autopsy kidneys showed low cross-reactivity (0.3 to 41.3% only) (Mannik et al. 2003). Moreover, about 15–26% of the antibodies in SLE are polyreactive between a diverse antigen panel that includes ssDNA, dsDNA, LPS, and insulin, pointing to an aberrant B-cell response (Mietzner et al. 2008). In the absence of ANA, dsDNA in serum, or IgG eluted from kidney homogenates, NZM2328.Lc4 mice developed glomerular deposits and fatal LN (Mortensen and Rekvig 2009). Therefore, development of LN is not associated with pathogenic autoantibodies of singular specificities and majority of the antigens recognized by glomerular IgG deposits are unknown.

Nevertheless, of all the nucleosomal antibodies in SLE, dsDNA antibodies are potentially the most specific and pathogenic. Electron dense deposits (EDS) were seen in proteinuric, lupus-susceptible NZB/W F1 mice (Mortensen and Rekvig 2009). These EDS were oligonucleosomes, containing chromatin fragments and nucleosomal DNA deposited on the glomerular basement membrane that were recognized by anti-dsDNA monoclonal antibodies. These oligonucleosomes were the result of ineffective fragmentation and clearance of apoptotic material in the renal glomerulus (Mortensen and Rekvig 2009). It was also shown that anti-dsDNA antibodies did not cross react with glomerular proteins and were specific for the oligonucleosomes (Mortensen and Rekvig 2009).

Recently, *PLA2R1* variants were shown to be associated with SLE in a Chinese population (Li et al. 2016). *PLA2R1* encodes the podocyte phospholipase A2 receptor (PLA2R) against which autoantibodies are found to be directed in membranous GN (Qin et al. 2011). We showed that in a large biopsy-proven European cohort, a missense variant (rs35771982) in *PLA2R1* strongly associated (*P* = 1.4 × 10^−14^) with PLA2R antibody-positive membranous GN (Saeed et al. 2014). It is likely to be the functional variant increasing PLA2R antigenicity index and interacting with *HLA-DQA1 (*Saeed et al. 2014 *)*. We also showed that PLA2R antibody-negative membranous GN was not associated. Moreover, *APOL1* variants were found to be associated with PLA2R-positive membranous GN (Larsen et al. 2014). Taken together, these findings link PLA2R antibody-positive membranous GN to SLE in a similar manner to MDA5 syndrome and AGS.

## Therapeutics

SLE treatment has so far largely been guided by clinical experience. With the recent advent of targeted biologic therapies, this paradigm has started to change. It only makes logical sense that the therapeutics be guided by disease pathogenesis, and some detailed studies evaluating the basic immunologic effects of traditional immunosuppressants have recently been published. In this section, we attempt to connect drug mechanisms with lupus pathobiology in the hope that this may provide insight into future treatment options.

In severe lupus flares, pulse therapy with methylprednisolone is instituted for 3–5 days which usually has a profound clinical effect on disease activity, albeit temporary. It was shown that methylprednisolone inhibited IGS and decreased IFN-α levels and pDC numbers in mice, whereas prednisolone did not. Interestingly, the effect of methylprednisolone lasted for about a week (Guiducci et al. 2010).

Hydroxychloroquine (HCQ) is a mandatory part of the SLE treatment regimen. HCQ affects the endosomal TLR activation by direct interaction with nucleic acid TLR ligands (Kuznik et al. 2011). HCQ formed complexes with nucleic acids in the endosomes leading to conformational changes in the nucleic acids, thereby preventing TLR activation (Kuznik et al. 2011). Similarly, TLR7 and TLR9 immunoregulatory sequences (IRS) injected in MRL/*lpr* mice improved the activity and chronicity indices for LN (Pawar et al. 2007). A bifunctional TLR 7/9 IRS blocked IFN-α production and PDC stimulation (Guiducci et al. 2010). Moreover, TLR7/9 signaling prevented glucocorticoid-induced apoptosis of PDCs and B-cells. Thus, continuous signaling of TLR 7/9 was responsible for the reduced efficacy of glucocorticoids at inhibiting the IFN pathway in SLE patients and lupus-prone mouse strains (Guiducci et al. 2010). Hence, inhibitors of TLR7/9 could prove to be effective corticosteroid-sparing drugs in SLE.

One of the mouse models used in this study was a TLR7-overexpressing transgenic mouse that developed a lupus-like syndrome (Subramanian et al. 2006). It is also known that TLR7 contributes specifically to anti-Sm-RNP antibodies (Christensen et al. 2006), and U1snRNP RNA was identified as an endogenous ligand for TLR7 (Savarese et al. 2006). Aberrant ssDNA processing by mutant *TREX1* may also stimulate TLR9 (Crow et al. 2006). Therefore, the endosomal TLR pathway is a potential therapeutic target for SLE of immense significance.

Cyclophosphamide (CYC) is an alkylating agent and is one of the most potent immunosuppressive drugs. It has a long history of use as induction therapy for organ-threatening SLE. CYC exerts its cytotoxic effect on predominantly B-lymphocytes by crosslinking DNA with alkyl groups, thus inhibiting DNA replication. Plasmablasts and plasma cells are not affected by cyclophosphamide (Miller and Cole 1967; Fassbinder et al. 2015); however, naïve B-cells are rapidly depleted (Fassbinder et al. 2015). Azathioprine (AZA) used in maintenance therapy for SLE, is a prodrug for 6-mercaptopurine, a purine analog impeding DNA synthesis via several enzymes including inosine monophosphate dehydrogenase (IMPDH). It has been shown to also substantially reduce transitional and naïve B-cells in peripheral blood of SLE patients (Eickenberg et al. 2012).

Mycophenolate mofetil (MMF) is used as an induction and maintenance regimen in SLE. MMF is a prodrug of mycophenolic acid which reversibly inhibits IMPDH, preferentially the type-II form of the enzyme that is upregulated in activated lymphocytes, leading to cell cycle arrest (Lee et al. 1985). Plasmablasts and plasma cells were significantly reduced in peripheral blood of MMF-treated patients with subsequent effect on antibody production (Fassbinder et al. 2015; Eickenberg et al. 2012). In contrast to CYC, T-cell subsets are unaffected by both MMF and AZA. CYC treatment was shown to increase peripheral blood CD8+ T-cells and pDCs (Fassbinder et al. 2015). Interestingly, SLE patients have low numbers of circulating pDCs likely thought to be due to tissue infiltration by these cells (Cederblad et al. 1998). It has been suggested that the rapid onset of action of CYC may be attributed to the more efficient inhibition of tissue inflammation leading to early reconstitution of circulating pDCs and CD8+ T-cells (Fassbinder et al. 2015).

Methotrexate (MTX) is often used to treat the arthritis in SLE; however, it has been shown to suppress overall SLE disease activity as well (Miyawaki et al. 2013; Sakthiswary and Suresh 2014). In an open-label study, low-dose MTX (7.5 mg) improved anti-dsDNA antibody, C3 and C4 levels, SLE disease activity, and had a steroid-sparing effect (Miyawaki et al. 2013). A meta-analysis also showed a substantial beneficial effect of MTX on SLE activity (Sakthiswary and Suresh 2014).

Targeted biologic therapy has revolutionized the treatment of SLE. Rituximab (RTX) is a chimeric monoclonal antibody against CD20 and affects B-cell subsets except plasma cells (Fernández-Nebro et al. 2012). Though RTX did not show superiority to conventional treatment regimens in SLE and LN in two randomized controlled trials (Rovin et al. 2012; Merrill et al. 2010), it has shown substantial benefit in several observational studies (Duxbury et al. 2013). It is postulated that lupus heterogeneity and perhaps stringent response criteria may have caused this discrepancy and further clinical trials are needed. It is important to note that RTX is far less toxic than CYC and therefore advantageous to use in SLE flares as induction therapy (Moroni et al. 2014).

Belimumab, approved for treatment of SLE, is a human monoclonal antibody targeting B-cell activating factor (BAFF), a TNF-like cytokine involved in B-cell survival and differentiation. BAFF overexpression led to B-cell expansion and a lupus-like syndrome in mice, whereas BAFF inhibition delayed lupus onset (Boneparth and Davidson 2012). Two large phase III Belimumab clinical trials in SLE showed that the therapeutic effect of belimumab lasted 1 year with reduction in SLE flares and steroid use; however, the onset was delayed and therefore not considered suitable for acute SLE (Boneparth and Davidson 2012). Belimumab decreased naïve, transitional B-cells and plasmablasts (Boneparth and Davidson 2012).

In contrast to murine studies mentioned above, CTLA4 blockade by Abatacept in randomized controlled trials did not improve outcomes of LN when used on a background of MMF/steroids or CYC/AZA (Furie et al. 2014; ACCESS Trial Group 2014). However, a small open-label study showed modest benefit in SLE and one of the LN trials showed that Abatacept improved anti-dsDNA antibody and C3 and C4 titres (Furie et al. 2014; Danion et al. 2016).

In a phase Ib randomized controlled trial of sifalimumab, a human anti-IFNα monoclonal antibody that specifically neutralizes most IFNα subtypes, SLE disease activity decreased in patients with high IGS at baseline in the combined sifalimumab group compared to placebo. Since this was a safety and dose study, the multiple sifalimumab dose groups made comparisons difficult and overall, there was no difference from placebo (Petri et al. 2013). A subsequent randomized, double-blind, placebo-controlled study of sifalimumab was barely significant (*P* = 0.053; though the a priori cutoff for this trial was unconventionally *P* = 0.1), and there was no clear dosage effect (Khamashta et al. 2016). Similarly, a phase II trial of another anti-IFN-α monoclonal antibody, rontalizumab, failed to meet its primary and secondary endpoints (Kalunian et al. 2016). However, recently, an IFN-α receptor monoclonal antibody, anifrolumab, significantly reduced SLE disease activity across multiple clinical global and organ-specific endpoints compared to placebo (Furie et al. 2015). SLE with high IRS responded better and it is hoped that IRS may evolve into a treatment prediction tool. Phase III trial of anifrolumab in SLE is underway and if the results hold, anifrolumab could be approved for treatment of SLE as well. The superior response of anifrolumab to anti-IFN-α protein monoclonals also strengthens the concept of cellular inhibition in SLE through receptor inhibition, rather than ameliorating cytokine responses.

## Summary

With now over a 100 known genes, lupus pathobiology has been transformed. It is clear that multiple immunologic pathways are activated and interact to lead to the clinical syndrome we recognize as SLE. From the vast genomic findings, few key features have emerged, summarized in Fig. 1. In essence, at the cellular level, pDC and B-cells appear to be the predominant cell types involved in SLE. T-cells may play coordinative (Th1) as well as partially cytotoxic (Th17) roles. Aberrant B-cell functioning is involved in both autoantibody production and ineffective IC clearance. The cycle of immunologic abnormalities may be triggered by foreign or self-antigens; however, the perpetuation of this cycle is of far greater significance and is due to a variety of genetic defects as detailed here.

In the near future, genomics tools may enhance the diagnostic capabilities of physicians taking care of SLE patients, just as nuclear antibodies and complement components do. The IGS has already been used in recent clinical trials (Petri et al. 2013; Kalunian et al. 2016). The challenge will be more robust clinical classification of SLE. As more antibodies are discovered, such as MDA5 and PLA2R, and genetic tests seep into clinical practice, it is likely that SLE classification may change further. Perhaps, SLE clinical definition may incorporate a mandatory presence of nuclear antibodies (ANA and its subsets) and the nuclear-antibody negative SLE may be classified according to the specific antibodies or mutation findings. In that case, PLA2R antibody-positive disease will be defined as a syndrome in its own right outside lupus and *IFIH1* mutation-positive disease will be classified under the MDA5 syndrome not associated with either dermatomyositis or SLE. On the other hand, expert panels may take a more inclusive approach by subclassifying SLE into more refined phenotypes. Whatever the course SLE classification takes in the future, genetic exploration of this complex disease has not only enhanced its own pathobiology but opened doors to understanding autoimmune disease in general as well.
